# Regular Exercise Is Associated with a Reduction in the Risk of NAFLD and Decreased Liver Enzymes in Individuals with NAFLD Independent of Obesity in Korean Adults

**DOI:** 10.1371/journal.pone.0046819

**Published:** 2012-10-22

**Authors:** Ji Cheol Bae, Sunghwan Suh, Se Eun Park, Eun Jung Rhee, Cheol Young Park, Ki Won Oh, Sung Woo Park, Sun Woo Kim, Kyu Yeon Hur, Jae Hyeon Kim, Myung-Shik Lee, Moon Kyu Lee, Kwang-Won Kim, Won-Young Lee

**Affiliations:** 1 Division of Endocrinology and Metabolism, Department of Internal Medicine, Samsung Medical Center, Sungkyunkwan University School of Medicine, Seoul, Republic of Korea; 2 Division of Endocrinology and Metabolism, Department of Internal Medicine, Kangbuk Samsung Hospital, Sungkyunkwan University School of Medicine, Seoul, Republic of Korea; University of Verona, Ospedale Civile Maggiore, Italy

## Abstract

**Background:**

We evaluated the association of regular physical exercise with the presence of non-alcoholic fatty liver disease (NAFLD) and liver enzymes in relation to obesity and insulin resistance.

**Methodology/Principal Findings:**

A cross-sectional analysis was conducted in 72,359 healthy Korean adults without diabetes who participated in a comprehensive health check-up. Subjects who have been exercising regularly (more than 3 times per week, at least for 30 minutes each time and for consecutive 3 month) were categorized into exercise group. All subjects were categorized into deciles based on their body mass index (BMI) and we estimated the odds ratios (ORs) for having NAFLD according to exercise regularity in each decile. The diagnosis of NAFLD was based on ultrasonography findings. Individuals with NAFLD (n = 19,921) were analyzed separately to evaluate ORs for having elevated liver enzymes based on regularity of exercise. The risk for NAFLD was significantly reduced in exercise group with age- and sex-adjusted ORs of 0.53–0.72 for all BMI deciles except at BMI categories of <19.6 and 20.7–21.6 kg/m^2^. While no difference was seen in BMI between subjects in exercise and non-exercise group across the BMI deciles, the values of body fat percentage and metabolic risk factors differed. Among NAFLD patients, subjects in exercise group had a lower risk for having elevated liver enzymes with multivariable adjusted OR of 0.85 (95% CI 0.74–0.99, for AST) and 0.74 (95% CI 0.67–0.81, for ALT) than did subjects in non-exercise group.

**Conclusions/Significance:**

Regular exercise was associated with a reduced risk for having NAFLD and decreased liver enzymes in patients with NAFLD, and this relationship was also independent of obesity.

## Introduction

Non-alcoholic fatty liver disease (NAFLD) encompasses liver conditions ranging from hepatic steatosis through steatohepatitis to cirrhosis [1]. Insulin resistance and obesity represent the most important risk factors for the development of NAFLD [2]. NAFLD is prevalent worldwide and affects ∼30% of adults [3]. Excess liver fat is an independent risk factor for cardiovascular disease, insulin resistance, pre-diabetes and type 2 diabetes (T2DM) [3], [4]. Rising prevalence of obesity and T2DM, particularly in younger people, will ensure that NAFLD remains a growing clinical concern for the future [5]. Despite evidence that physical inactivity can activate pathologies including insulin resistance and central adiposity which are closely linked to NAFLD [6], studies confirming this mechanistic link are lacking. To date, the role of physical activity for NAFLD has been tested in only a few studies with small numbers or male patients [7]. The present study is a large-scale cross-sectional study to explore the association of regular physical exercise, in relation to obesity and insulin resistance, with the presence of NAFLD and liver enzymes in subjects with NAFLD.

## Methods

### Subjects

A cross-sectional analysis was conducted among healthy Koreans without diabetes. Initial data were obtained from 92,205 subjects aged over 20 years who participated in a comprehensive health check-up from January to December 2008 at Kangbuk Samsung Hospital Total Healthcare Center. Among these subjects, 19,846 individuals with any of the following were excluded: (1) alcohol intake >30 g per day ( *n* = 12,069), (2) positive serologic markers for hepatitis B ( *n* = 3,698) or C ( *n* = 279) virus, (3) with known diabetes mellitus ( *n* = 2,971), fasting plasma glucose concentration ≥126 mg/dl ( *n* = 2,628) or HbA1c ≥6.5% ( *n* = 2,605), (4) abnormal ultrasonographic findings (i.e. liver cirrhosis, suspected hepatocellular carcinoma, hepatic mass, or signs of *Clonorchis sinensis*) of the liver ( *n* = 271), (5) a history of malignancy ( *n* = 1,352), (6) hyper- or hypothyroidism (*n* = 513), or (7) absence of data in the questionnaire, anthropometric measurements, and HbA1c levels (*n* = 1,198, *n* = 559, *n* = 134, respectively). After applying the above exclusion criteria, the total number of subjects eligible for the study was 72,359 (38,970 men and 33,389 women with a mean age of 42.0 years). Informed consent requirement for this study was exempted by the institutional review board because researchers only accessed the database for analysis purposes, and personal information was not accessed. This study was approved by the institutional review board at Kangbuk Samsung Hospital.

### Measurements

Subjects' heights and body weights were measured barefoot wearing light clothing. Body mass index (BMI) was calculated as weight in kilograms divided by the square of the height in meters. Percentage body fat was estimated using multi-frequency bioimpedance analyzer (InBody 720, Biospace Co., Seoul, Korea) which was validated with regards to reproducibility and accuracy for body composition [8], with eight point tactile electrodes (2 for each foot and hand). Participants' percentage body fat was automatically calculated with the calculated whole body and segmental bioelectrical impedance by using the manufacturer's equations. Blood samples were collected from the antecubital vein after an overnight fast. Fasting blood glucose, total cholesterol (TC), triglyceride (TG), low-density lipoprotein cholesterol (LDL-C), high-density lipoprotein cholesterol (HDL-C), alanine aminotransferase (ALT), and aspartate aminotransferase (AST) were measured using Bayer Reagent Packs on an automated chemistry analyzer (Advia 1650 Autoanalyzer, Bayer Diagnostics, Leverkusen, Germany). As a marker of insulin resistance, the homeostatic model assessment of insulin resistance (HOMA-IR) was calculated using the following formula [9]: HOMA-IR  =  (fasting insulin ( μ IU/ml) × fasting glycemia (mmol/l))/22.5.

Abdominal ultrasonography (Logic Q700 MR, GE, Milwaukee, WI, U.S.A.) was performed in all subjects, and fatty liver was diagnosed based on known standard criteria, including hepatorenal echo contrast, liver brightness, and vascular blurring, using a 3.5 MHz probe [10]. Several experienced radiologists, all of whom were blinded to the clinical statuses of the subjects, performed ultrasounds. Participants were informed of their ultrasound results only after they completed their questionnaires.

### Physical exercise

In this study, physical exercise was assessed by self-reported questionnaires listed in supplementary Table 1, which included questions about duration, frequency, and intensity of exercise. Regular exercise was defined as doing physical exercise of at least moderate intensity more than 3 times per week, for at least 30 minutes each time, for an uninterrupted duration of at least 3 month at the time of the questionnaire. Physical activity of moderate intensity is defined as requiring a metabolic equivalent task (MET) score of 3.0–6.0 and a typical activity of moderate intensity is “brisk” walking at 5.6 km/h (3.5 miles/h) on a flat surface requiring 3.8 MET [11]. In our study, moderate exercise was defined by activity leading to breathing relatively harder than normal or puff and pant such as brisk walking, climbing, swimming, bicycling at regular pace, badminton, dancing or other exercise in the questionnaire.

**Table 1:** Clinical characteristics based on the exercise regularity.

	Non-exercise	Exercise		All
	(n = 59,392)	(n = 12,967)	*P* value	(N = 72,359)
Male (%)	32,447 (54.6)	6,507 (50.2)	<0.001*	38,970 (53.8)
Age (years)	41.3±8.3	45.2±8.9	<0.001†	42.0±8.5
BMI (kg/m^2^)	23.3±3.1	23.6±2.8	<0.001†	23.4±3.0
Body fat (%)	24.7±6.1	24.7±6.1	0.903†	24.7±6.1
Male	21.7±4.8	21.1±4.5	<0.001†	21.5±4.7
Female	28.4±5.6	28.4±5.3	0.351†	28.4±5.5
Fasting glucose (mg/dl)	93.1±8.7	93.3±9.1	0.082†	93.1±8.5
HbA1c (%)	5.37±0.29	5.38±0.29	<0.001†	5.37±0.29
HOMA-IR	1.90±0.93	1.87±0.85	<0.001†	1.90±0.92
Total-cholesterol (mg/dl)	194.4±33.5	196.8±32.9	<0.001†	194.8±33.4
Triglyceride (mg/dl)	122.2±79.5	111.0±66.7	<0.001†	120.2±77.5
LDL-C (mg/dl)	110.5±29.3	110.7±28.6	0.436†	110.6±29.2
HDL-C (mg/dl)	55.2±12.7	57.6±13.4	<0.001†	55.6±12.8
Male	50.6±10.6	52.6±11.2	<0.001†	50.9±10.9
Female	60.7±13.3	62.6±13.7	<0.001†	61.0±13.2
Systolic BP (mmHg)	112.8±13.6	114.5±14.2	<0.001†	113.1±13.7
AST (unit/l)	23.7±10.3	24.4±9.8	<0.001†	23.8±10.2
ALT (unit/l)	25.1±18.9	23.2±13.8	<0.001†	24.8±18.1
Current smoking (%)	12,909 (21.7)	1,800 (13.9)	<0.001*	14,709 (20.3)
NAFLD (%)	16,873 (28.4)	3,048 (23.5)	<0.001*	19,921 (27.5)

BMI, body mass index; HOMA-IR, homeostasis model assessment of insulin resistance; LDL-C, low-density lipoprotein cholesterol; HDL-C, high-density lipoprotein cholesterol; BP, blood pressure; AST, aspartate aminotransferase; ALT, alanine aminotransferase; NAFLD, non-alcoholic fatty liver disease.

Data are numbers (%) or means ± standard deviation.

* By Pearson's χ^2^ –test.

† By unpaired *t –*test.

### Study design and statistic analysis

All 72,359 subjects were categorized into deciles based on the BMI level with cut-points of 19.6, 20.7, 21.6, 22.4, 23.2, 24.0, 24.8, 25.8, and 27.8 kg/m^2^. Also, all subjects were categorized into exercise and non-exercise group according to whether they have been exercising regularly or not. We estimated the odds ratios (ORs) for having NAFLD according to exercise regualrity in BMI deciles respectively using a logistic regression model. Two-way analysis of variance was used to evaluate difference in values of metabolic risk factors between exercise and non-exercise group across the BMI deciles. Among all participants, individuals with NAFLD (n = 19,921) proven by ultrasonography were analyzed separately to evaluate ORs for having a high liver enzyme based on the regularity of exercise using a logistic regression model. A serum ALT >41 units/l or AST >38 units/l were defined as having elevated liver enzymes [12]. Results are expressed as subject number with percentage (%) and mean value with standard deviation. The unpaired Student's *t -*test and Pearson's χ^2^-test was used to analyze statistical differences in the characteristics of the study participants between exercise and non exercise groups. During analysis, missing values of variables were replaced with mean values and the highest number for missing value was 238 (0.33%) seen in the variable of HOMA-IR. Statistical data analysis was performed using the SPSS program, version 17.0 (SPSS, Chicago, IL, USA). All the reported *P* values are two-tailed, and statistical significance was set at *p*<0.05.

## Results

**Figure 1:**
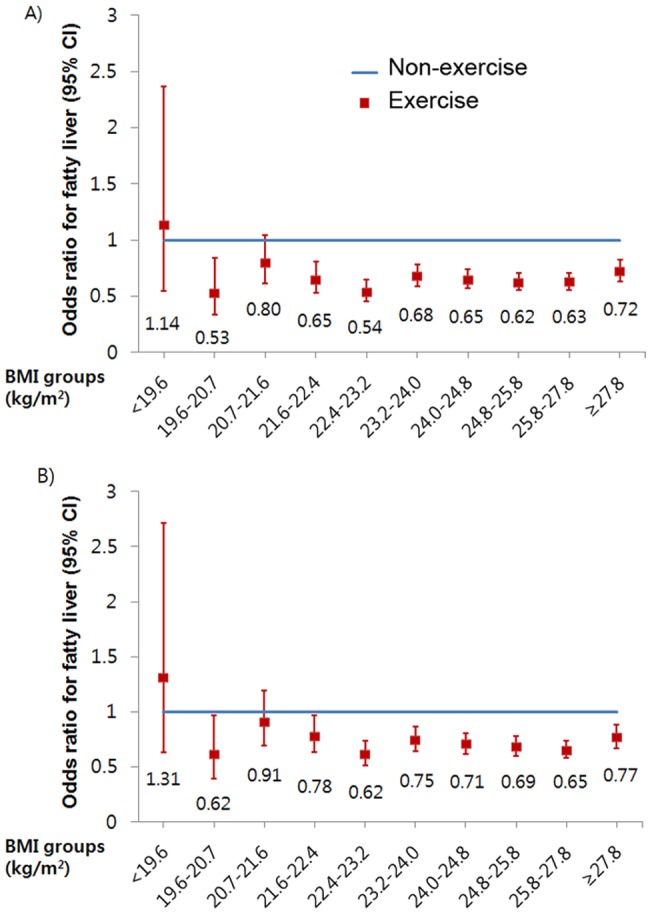
The risk for NAFLD by exercise regularity analyzed separately according to the BMI deciles. **A) Adjusted for age and sex. B) Adjusted for age, sex, and body fat percentage. Odds ratios were estimated from binary logistic regression analysis.**

**Figure 2:**
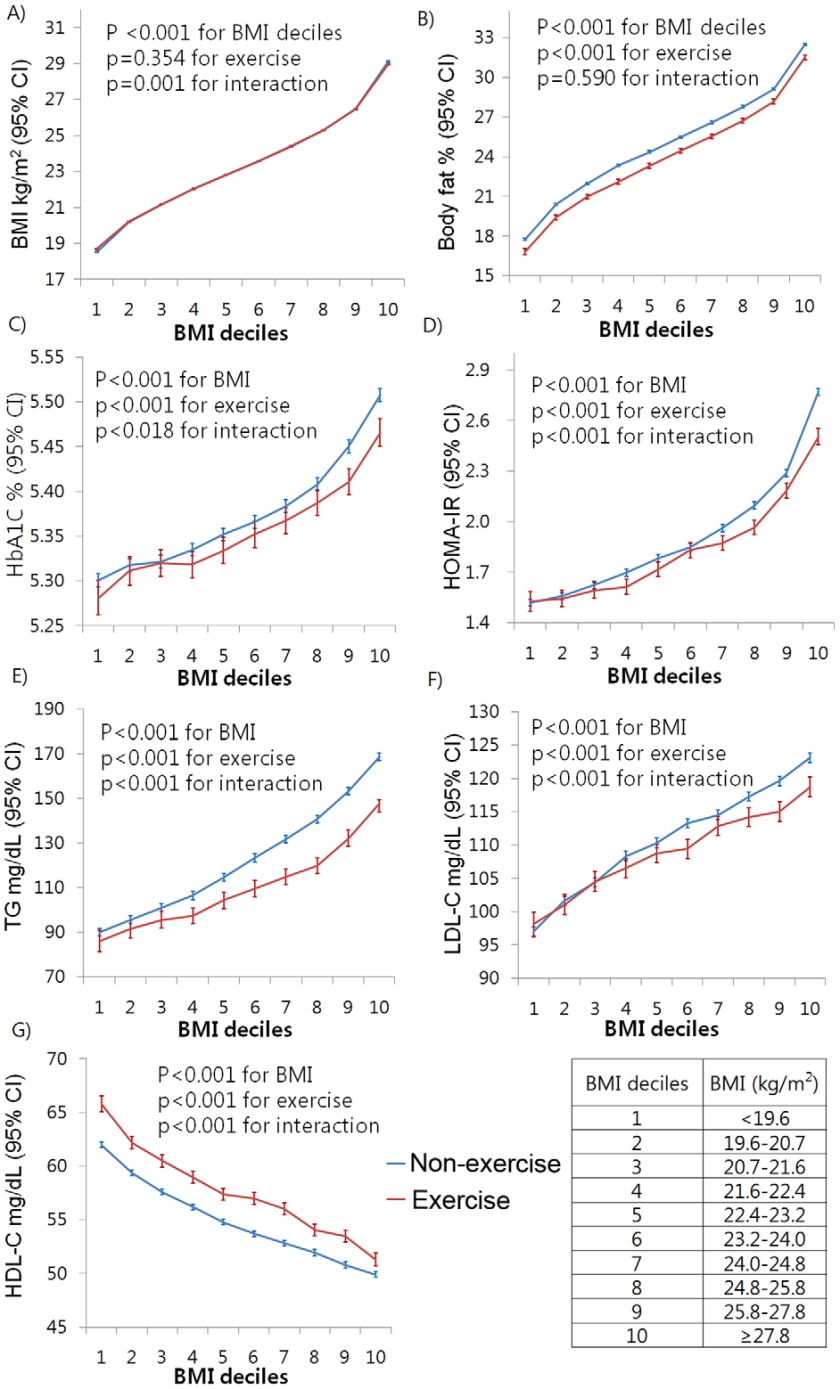
Association of metabolic risk factors with exercise regularity across the BMI level. Adjusted for age and sex.

The participants were relatively young and not obese with a mean age of 42.0±8.5 and BMI of 23.4±3.0 kg/m^2^. We found that 27.5% of non-diabetic subjects had NAFLD, and the prevalence of NAFLD was lower (23.5% vs. 28.4%) in exercise group. 17.9% among all subjects were exercising regularly, and they were older (45.2±8.9 vs. 41.3±8.3) and had higher BMI (23.6±2.8 vs. 23.3±3.1) than subjects in non-exercise group (Table 1). The prevalence of NAFLD increased steadily with increasing BMI level in both exercise and non-exercise group (Table 2). When the risk for NAFLD was analyzed separately according to BMI level, the risk was reduced significantly in exercise group with age- and sex-adjusted ORs of 0.53–0.72 for all BMI deciles except at BMI groups of <19.6 and 20.7–21.6 kg/m^2^ (Figure 1A). No difference was seen in BMI between exercise and non-exercise groups across the BMI deciles (*p* = 0.354 for physical activity, Figure 2A). This reduced risk still remained significant after additional adjustment for body fat percentage (Figure 1B). The values of body fat percentage, HOMA-IR, HbA1c, triglyceride, and LDL-C also increased steadily with an increasing BMI level and those values differed between subjects in exercise and non-exercise group across the BMI categories (*p*<0.001 for physical activity, Figure 2, Suppl Figure 1). There was significant interaction between BMI and exercise habit on the levels of HbA1C (*p* = 0.018), LDL-C (*p<*0.001), TG (*p<*0.001), and HOMA-IR (*p<*0.001), while interaction on body fat percentage was not observed (*p* = 0.590 for interaction, Figure 2). Particularly, within each BMI decile, the prevalence of NAFLD differed according to the frequency (Suppl Table 2), duration (Suppl Table 3), and intensity (Supple Table 4) of exercise. When individuals with NAFLD were analyzed separately, subjects in exercise group had a lower risk for having elevated liver enzymes with age- and sex-adjusted OR of 0.75 (95% CI 0.64–0.88, for AST) and 0.66 (95% CI 0.61–0.73, for ALT) than did subjects in non-exercise group. Even after additional adjustment for body fat percentage, triglyceride, LDL, HDL, systolic BP, HOMA-IR and smoking, this reduced OR remained significant (Table 3).

**Table 2:** The prevalence of NAFLD according to the exercise group and BMI deciles.

BMI category	Non-exercise			Exercise		
(N = 72,359)	Non-NAFLD	NAFLD	n = 59,392	Non-NAFLD	NAFLD	N = 12,967
<19.6 (7,243)	6,393	53 (0.8)	6,446	788	9 (1.1)	797
19.6–20.7 (7,284)	5,919	201 (3.3)	6,120	1,142	22 (1.9)	1,164
20.7–21.6 (7,194)	5,481	373 (6.4)	5,854	1,268	72 (5.4)	1,340
21.6–22.4 (7,291)	5,183	711 (12.1)	5,894	1,281	116 (8.3)	1,397
22.4–23.2 (7,170)	4,588	1,182 (20.5)	5,770	1,228	172 (12.3)	1,400
23.2–24.0 (7,247)	4,263	1,579 (27.0)	5,841	1,122	284 (20.2)	1,406
24.0–24.8 (7,241)	3,696	2,111 (36.4)	5,807	1,038	396 (27.6)	1,434
24.8–25.8 (7,267)	3,069	2,745 (47.2)	5,814	929	524 (36.1)	1,453
25.8–27.8 (7,204)	2,433	3,418 (58.4)	5,851	716	637 (47.1)	1,353
≥27.8 (7,218)	1,494	4,501 (75.1)	5,995	407	816 (66.7)	1,223

NAFLD, non-alcoholic fatty liver disease; BMI, body mass index.

Data are numbers (%).

**Table 3:** Odds ratios for AST>41, ALT>38 IU according to the exercise regularity in subjects with NAFLD.

	NAFLD (n = 19,921)		
Variables	Non-exercise	Exercise	*P* value
Subjects, n	16,873	3,048	
Male, No. (%)	13,573 (80.4)	2,262 (74.2)	
Subjects with AST>41unit/l, No. (%)	1,431 (8.5)	192 (6.3)	
Subjects with ALT>38unit/l. No. (%)	5,947 (35.2)	737 (24.2)	
Adjusted odds ratio for AST >41 unit/l (95% CI)*			
Age and sex	1	0.75 (0.64–0.88)	<0.001
Age, sex, and body fat percentage	1	0.80 (0.68–0.93)	0.007
Age, sex, body fat percentage, TG, LDL, HDL	1	0.85 (0.74–0.99)	0.042
systolic BP, HOMA-IR, and smoking			
Adjusted odds ratio for ALT >38 unit/l (95% CI)*			
Age and sex	1	0.66 (0.61–0.73)	<0.001
Age, sex, and body fat percentage	1	0.70 (0.64–0.77)	<0.001
Age, sex, body fat percentage, TG, LDL, HDL	1	0.74 (0.67–0.81)	<0.001
systolic BP, HOMA-IR, and smoking			

NAFLD, non-alcoholic fatty liver disease; AST, aspartate aminotransferase; ALT, alanine aminotransferase; BP,

Blood pressure; TG, triglyceride; LDL-C, low-density lipoprotein cholesterol; HDL-C, high-density lipoprotein.

Cholesterol; HOMA-IR, homeostasis model assessment of insulin resistance.

* Estimated by binary logistic regression analysis.

## Discussion

In this large cross-sectional study of non-diabetic subjects, regular physical exercise was associated with a reduced risk for having NAFLD and this risk reduction was shown through all BMI categories except at the very low group (BMI <19.6 kg/m^2^). In particular, the finding that no difference was seen in BMI between subjects in exercise and non-exercise group across the BMI deciles is noticeable, and that finding is also supported by the results in some studies showing that exercise does not need to reduce body weight to have a beneficial effect on improving hepatic lipid metabolism and reducing hepatic fat [13]–[15]. It is not certain whether weight reduction was observed in subjects who exercise regularly before taking a health check-up in our study. However, weight loss is difficult to achieve in clinical practice, and a previous meta-analysis of the effects of exercise has shown that exercise intervention produced no statistically significant reduction in body weight [16]. Also, in our study, given criterion defined as regular exercise was lower in intensity compared to those in other exercise intervention study (16).

Insulin resistance leading to dysregulated energy metabolism between adipose tissue, skeletal muscle and the liver is a major feature of NAFLD and contributes to hepatic steatosis [17], [18]. In the present study, values of HOMA-IR and prevalence of NAFLD increased steadily with an increasing BMI level. However, these values differed between exercise and non-exercise group across the BMI categories and similar findings were also shown in other metabolic constituents such as HbA1c, triglyceride, LDL-C, and HDL-C. Individuals who did regular exercise were less insulin resistant, despite having the same BMI, than those who did not. This result may help explain different risks for having NAFLD according to exercise habit among subjects with the same BMI.

Subjects in exercise group showed a lower percentage of body fat compared with subjects in non-exercise group across the BMI categories, whereas no differences were seen for BMI. These results are in accordance with those of previous studies that increasing physical activity can result in an increase in lean body mass and that at any given weight, individuals who exercise more have less visceral fat than those who are sedentary [19], [20]. The difference shown in body fat percentage may have contributed to the difference in insulin resistance and NAFLD risk between subjects in exercise and non-exercise group [21]. Meanwhile, it is interesting that there was significant interaction between BMI and exercise habit on the levels of HbA1c, LDL-C, TG, and HOMA-IR, while interaction on the body fat percentage was not seen, indicating that more obese individuals have a better metabolic response to physical exercise. Along with the result of a sustained risk reduction for having NAFLD after additional adjustment for body fat percentage, our data also suggest that regular physical exercise has an association with insulin resistance independent of body fat percentage. A recent study demonstrated that exercise alone enhances insulin sensitivity independent of change in body composition [22] and this independent effect of exercise on insulin resistance is explained through a number of mechanisms [16].

Kistler and colleagues have shown that vigorous but not moderate exercise, nor total duration or volume of physical activity, is related to decreased odds of having NASH [23]. However, in our study, reduced risk for having NAFLD was associated with the not only intensity, but also duration and frequency of physical exercise. Regular exercise with sufficient duration might be associated with reduced risk for NAFLD, despite of moderate intensity.

Although aminotransferase levels are not confidently used as surrogate markers of the severity of steatosis and necroinflammatory infiltration, significant differences in liver enzyme levels were reported between pure fatty liver and non-alcoholic steatohepatitis (NASH) proven by liver biopsy [24], [25]. Lifestyle interventions incorporating weight loss improve the histopathological changes seen in NAFLD and NASH, which is accompanied by an improvement in liver enzyme [26], [27]. Chronic liver diseases affecting the liver enzyme and subjects with alcohol consumption were excluded in our examination. Thus, among subjects with NAFLD, a different risk for having elevated liver enzymes based on their exercise habit may reflect the difference in severity of NAFLD between exercise and non-exercise group. Interestingly, this different risk still remained significant even after adjustment for HOMA-IR, body fat percentage and other traditional risk factors, which suggests that direct association of exercise with liver fat may exist, beyond insulin resistance [3], [28]. Emerging evidence indicates that exercise may also modulate hepatic fat by directly altering hepatic lipid oxidation and lipogenesis [29]. Mitochondria is the primary site for the oxidation of fatty acids and hepatocytes are rich in mitochondria [30]. AMPK activity is increased during exercise in rodents and is known to increase hepatic fatty acid b-oxidation within mitochondria [31], [32].

Even though the majority of evidence supports a beneficial effect of exercise on liver fat [33], discordant result between exercise and NAFLD was also reported [34]. These discordant results may be at least partly related to unmeasured confounders such diet or medication.

Our results may not be directly applied to western population because BMI cut-off value for obesity in Koreans is different from Western. In Asian, BMI greater than 23 kg/m^2^ is considered overweight and above 25 kg/m^2^ is considered obese corresponding with 25 kg/m^2^ and 30 kg/m^2^ in western people, respectively [35]. In our study population, mean BMI was 23.4 kg/m^2^ and 52% had BMI greater than 23 kg/m^2^.

A limitation of our study is that self-reported information might be prone to recall and social desirability bias. In addition, our data could not establish a causal relationship between regular exercise and NAFLD because of the cross-sectional design of this study. The use of ultrasonography to diagnose fatty liver is also another limitation. Although ultrasonography is reasonably accurate, it cannot identify fatty infiltration of the liver below a threshold of 30% [36]. We do not have sufficient dietary and medication data on this group of participants. Diet composition influences liver fat content and regular exercisers may have healthier dietary habit than non-exercisers [37], [38]. Finally, we did not consider non-exercise physical activities such as occupational activity, which might have effect on the results.

Among subjects with the same BMI, individuals who exercised regularly were less insulin resistant and had a lower risk of having NAFLD. In addition, this finding was independent of body fat percentage. In individuals with NAFLD demonstrated by ultrasonography, the risk of having elevated levels of liver enzymes was lower in subjects who exercised regularly and this reduced risk was independent of obesity and insulin resistance. In conclusion, regular physical exercise was associated with a reduced risk for having NAFLD and decreased liver enzymes in patients with NAFLD, and this relationship was also independent of obesity.

## Supporting Information

Figure S1 **Association of HDL-C with exercise regularity across the BMI level. A) Male. B) Female.** Adjusted for age.
(TIF)

Table S1 **Questionnaire which was asked to subjects.**
(DOC)

Table S2 **The Odds ratio for NAFLD analyzed by the frequency of exercise according to the BMI deciles.**
(DOC)

Table S3 **The Odds ratio for NAFLD analyzed by the duration of exercise according to the BMI deciles.**
(DOC)

Table S4 **The Odds ratio for NAFLD analyzed by the intensity of exercise according to the BMI deciles.**
(DOC)
