# Supplementary figures and images for: Regular Exercise Is Associated with a Reduction in the Risk of NAFLD and Decreased Liver Enzymes in Individuals with NAFLD Independent of Obesity in Korean Adults
Source: PLoS One. 2012 Oct 22;7(10):e46819. doi: 10.1371/journal.pone.0046819 (PMC3478288; doi:10.1371/journal.pone.0046819)

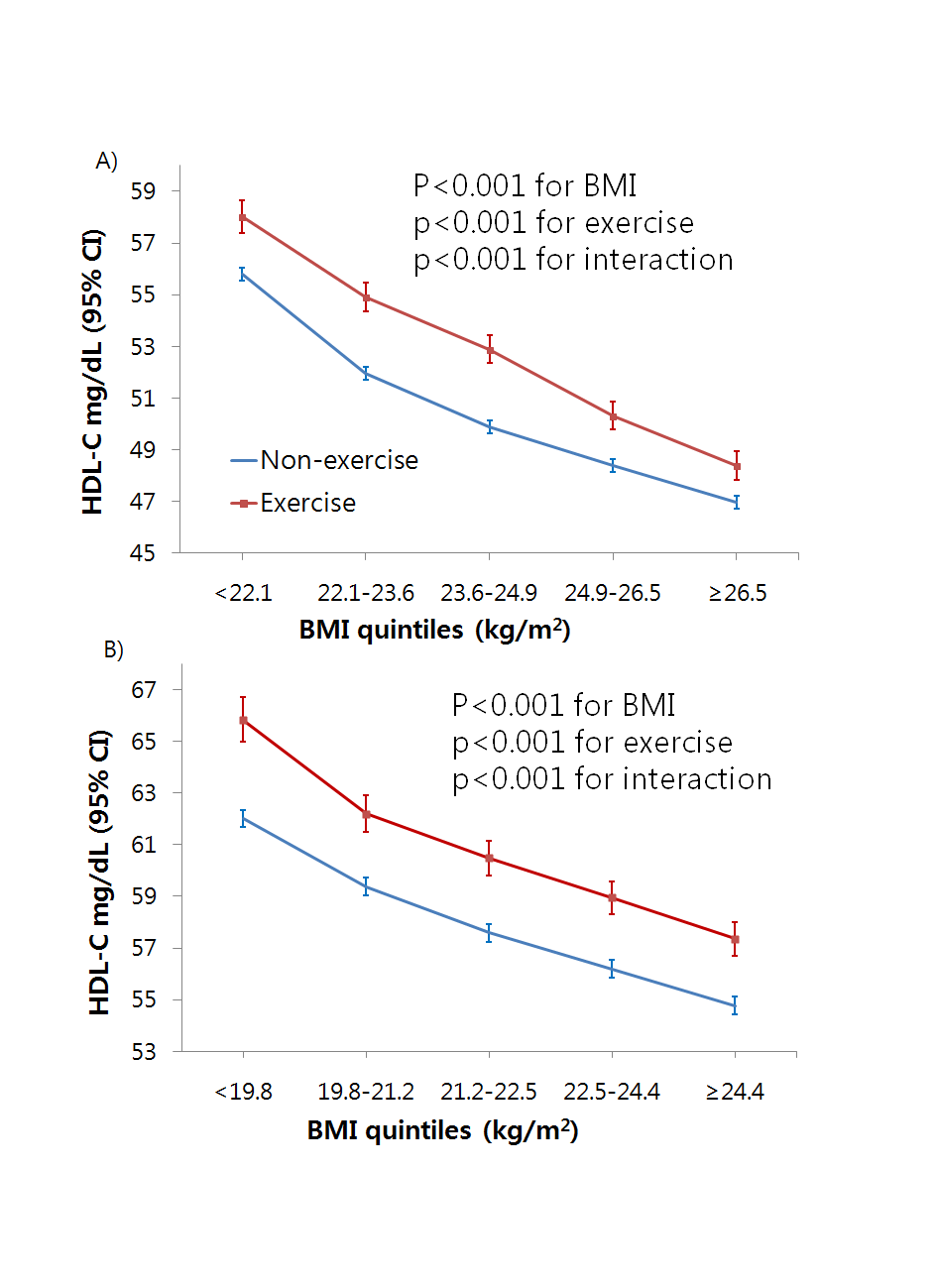

Supplement: Figure S1 — Association of HDL-C with exercise regularity across the BMI level. A) Male. B) Female. Adjusted for age. (TIF) [file pone.0046819.s001.tif]
