# Supplementary material for: Regular Exercise Is Associated with a Reduction in the Risk of NAFLD and Decreased Liver Enzymes in Individuals with NAFLD Independent of Obesity in Korean Adults
Source: PLoS One. 2012 Oct 22;7(10):e46819. doi: 10.1371/journal.pone.0046819 (PMC3478288; doi:10.1371/journal.pone.0046819)
Supplement: Table S1 — Questionnaire which was asked to subjects. (DOC) [file pone.0046819.s002.doc]

**Table S1. Questionnaire which was asked to subjects**

| Think about the physical exercise that you did in the last 1 month |
| --- |
| 1.During the last 1month, did you do regular physical exercise outside of work |
| a) yes, I did b) no, I didn’t |
| If you marked "a) yes I did", we suggest that you proceed to answer question 2 to 5 |
| 2. How many days did you do physical exercise usually per week? |
| a) 1-2times b) 3-4 times c) 5-6 times d) most days of week |
| 3. How much total time did you usually spend doing physical exercise on one of those days? |
| a) less than 30 minutes b) 30 minutes to 60 minutes c) more than 60 minutes |
| 4. How is intensity usually each time? |
| a) light activity not making you breath harder than normal |
| ex) walking, fishing, golf, bowling, less strenuous home exercise, calisthenics |
| b) moderate activity making you breath somewhat harder than normal |
| ex) brisk walking, climbing, swimming, bicycling at regular pace, badminton, dancing |
| c) vigorous activity making you breath harder than normal |
| ex) jogging, running, aerobics, tennis, vigorous bicycling, soccer |
| 5. How long have you been lasting doing regular physical exercise? |
| a) 1 to 3 months b) 4 to 6 months c) 6 to 12 months d) more than 1 year |
