# Supplementary material for: Regular Exercise Is Associated with a Reduction in the Risk of NAFLD and Decreased Liver Enzymes in Individuals with NAFLD Independent of Obesity in Korean Adults
Source: PLoS One. 2012 Oct 22;7(10):e46819. doi: 10.1371/journal.pone.0046819 (PMC3478288; doi:10.1371/journal.pone.0046819)
Supplement: Table S2 — The Odds ratio for NAFLD analyzed by the frequency of exercise according to the BMI deciles. (DOC) [file pone.0046819.s003.doc]

**Table S2. The Odds ratio for NAFLD analyzed by frequency of exercise according to the BMI deciles**

| BMI category | Non-exercise | | Exercise^＊^(n=12,967) | | | | OR^†^ (95% CI) | | |
| --- | --- | --- | --- | --- | --- | --- | --- | --- | --- |
| (n=72,359) | (n=59,392) | | 3-4 times per week | | ≥ 5times per week | | Non- | 3-4 times per | ≥ 5 times per |
|  | NAFLD | total | NAFLD | total | NAFLD | total | exercise | week | week |
| < 19.6 (7,243) | 53 (0.8) | 6,446 | 9 (1.6) | 553 | 0 (0) | 244 | 1 | 1.70 (0.83-3.50) |  |
| 19.6-20.7 (7,284) | 201 (3.3) | 6,120 | 14 (1.7) | 809 | 8 (2.3) | 355 | 1 | 0.51 (0.29-0.88) | 0.58 (0.28-1.20) |
| 20.7-21.6 (7,194) | 373 (6.4) | 5,854 | 58 (6.2) | 934 | 14 (3.4) | 406 | 1 | 0.94 (0.69-1.25) | 0.51 (0.29-0.89) |
| 21.6-22.4 (7,291) | 711 (12.1) | 5,894 | 83 (8.8) | 947 | 33 (7.3) | 450 | 1 | 0.67 (0.52-0.86) | 0.59 (0.41-0.85) |
| 22.4-23.2 (7,170) | 1,182 (20.5) | 5,770 | 121 (12.6) | 958 | 51 (11.5) | 442 | 1 | 0.56 (0.46-0.69) | 0.49 (0.36-0.66) |
| 23.2-24.0 (7,247) | 1,578 (27.0) | 5,841 | 213 (21.6) | 986 | 71 (16.9) | 420 | 1 | 0.72 (0.61-0.85) | 0.54 (0.42-0.71) |
| 24.0-24.8 (7,241) | 2,111 (36.4) | 5,807 | 292 (28.9) | 1,012 | 104 (24.6) | 422 | 1 | 0.68 (0.59-0.79) | 0.57 (0.45-0.72) |
| 24.8-25.8 (7,267) | 2,745 (47.2) | 5,814 | 382 (37.8) | 1,010 | 142 (32.1) | 443 | 1 | 0.67 (0.58-0.77) | 0.53 (0.42-0.65) |
| 25.8-27.8 (7,204) | 3,418 (58.4) | 5,851 | 489 (50.5) | 969 | 148 (38.5) | 384 | 1 | 0.70 (0.61-0.81) | 0.45 (0.37-0.56) |
| ≥27.8 (7,218) | 4,501 (75.1) | 5,995 | 602 (69.0) | 873 | 214 (61.1) | 350 | 1 | 0.76 (0.65-0.88) | 0.62 (0.49-0.78) |
|  | 16,873 (28.4) | 59,392 | 2,263 (25.0) | 9,051 | 785 (20.0) | 3,916 | 1 |  |  |

NAFLD, non-alcoholic fatty liver disease; BMI, body mass index

Data are numbers (%)

＊defined as doing physical exercise of at least moderate intensity more than 3 times per week, for at least 30 minutes each time, for an uninterrupted duration of at least 3 month at the time of the questionnaire

†Adjusted for age and sex. Estimated by binary logistic regression analysis
