# Supplementary material for: Regular Exercise Is Associated with a Reduction in the Risk of NAFLD and Decreased Liver Enzymes in Individuals with NAFLD Independent of Obesity in Korean Adults
Source: PLoS One. 2012 Oct 22;7(10):e46819. doi: 10.1371/journal.pone.0046819 (PMC3478288; doi:10.1371/journal.pone.0046819)
Supplement: Table S3 — The Odds ratio for NAFLD analyzed by the duration of exercise according to the BMI deciles. (DOC) [file pone.0046819.s004.doc]

**Table S3. The Odds ratio for NAFLD analyzed by the duration of exercise according to the BMI deciles**

| BMI category | Non-exercise | | Exercise^＊^(n=12,967) | | | | OR^†^ (95% CI) | | |
| --- | --- | --- | --- | --- | --- | --- | --- | --- | --- |
| (n=72,359) | (n=59,392) | | Duration < 1 year | | Duration ≥ 1 year | | Non- | Duration | Duration |
|  | NAFLD | total | NAFLD | total | NAFLD | total | exercise | < 1 year | ≥ 1year |
| < 19.6 (7,243) | 53 (0.8) | 6,446 | 6 (1.2) | 480 | 3 (0.9) | 317 | 1 | 1.44 (0.61-3.39) | 0.75 (0.23-2.50) |
| 19.6-20.7 (7,284) | 201 (3.3) | 6,120 | 9 (1.4) | 648 | 13 (2.5) | 516 | 1 | 0.43 (0.22-0.85) | 0.64 (0.36-1.15) |
| 20.7-21.6 (7,194) | 373 (6.4) | 5,854 | 44 (6.3) | 701 | 28 (4.4) | 639 | 1 | 1.07 (0.77-1.48) | 0.57 (0.38-0.86) |
| 21.6-22.4 (7,291) | 711 (12.1) | 5,894 | 68 (9.0) | 754 | 48 (7.5) | 643 | 1 | 0.77 (0.59-1.01) | 0.52 (0.38-0.71) |
| 22.4-23.2 (7,170) | 1,182 (20.5) | 5,770 | 110 (14.4) | 765 | 62 (9.8) | 635 | 1 | 0.71 (0.57-0.88) | 0.37 (0.28-0.49) |
| 23.2-24.0 (7,247) | 1,578 (27.0) | 5,841 | 177 (23.2) | 764 | 107 (16.7) | 642 | 1 | 0.85 (0.71-1.02) | 0.48 (0.38-0.60) |
| 24.0-24.8 (7,241) | 2,111 (36.4) | 5,807 | 249 (31.0) | 803 | 147 (23.3) | 631 | 1 | 0.80 (0.68-0.94) | 0.48 (0.39-0.59) |
| 24.8-25.8 (7,267) | 2,745 (47.2) | 5,814 | 286 (37.7) | 759 | 238 (34.3) | 694 | 1 | 0.71 (0.60-0.83) | 0.54 (0.45-0.64) |
| 25.8-27.8 (7,204) | 3,418 (58.4) | 5,851 | 409 (50.3) | 813 | 228 (42.2) | 540 | 1 | 0.72 (0.62-0.84) | 0.49 (0.41-0.59) |
| ≥27.8 (7,218) | 4,501 (75.1) | 5,995 | 548 (68.6) | 799 | 268 (63.2) | 424 | 1 | 0.76 (0.64-0.90) | 0.64 (0.52-0.80) |
|  | 16,873 (28.4) | 59,392 | 1,906 (26.2) | 7,286 | 1,142 (20.1) | 5,681 | 1 |  |  |

NAFLD, non-alcoholic fatty liver disease; BMI, body mass index

Data are numbers (%)

＊defined as doing physical exercise of at least moderate intensity more than 3 times per week, for at least 30 minutes each time, for an uninterrupted duration of at least 3 month at the time of the questionnaire

†Adjusted for age and sex. Estimated by binary logistic regression analysis
