# Supplementary material for: Regular Exercise Is Associated with a Reduction in the Risk of NAFLD and Decreased Liver Enzymes in Individuals with NAFLD Independent of Obesity in Korean Adults
Source: PLoS One. 2012 Oct 22;7(10):e46819. doi: 10.1371/journal.pone.0046819 (PMC3478288; doi:10.1371/journal.pone.0046819)
Supplement: Table S4 — The Odds ratio for NAFLD analyzed by the intensity of exercise according to the BMI deciles. (DOC) [file pone.0046819.s005.doc]

**Table S4. The Odds ratio for NAFLD analyzed by the intensity of exercise according to the BMI deciles**

| BMI category | Non-exercise | | Exercise^＊^(n=12,967) | | | | OR^†^ (95% CI) | | |
| --- | --- | --- | --- | --- | --- | --- | --- | --- | --- |
| (n=72,359) | (n=59,392) | | Moderate | | Vigorous | |  |  |  |
|  | NAFLD | total | NAFLD | total | NAFLD | total | Non-exercise | Moderate | Vigorous |
| < 19.6 (7,243) | 53 (0.8) | 6,446 | 9 (1.3) | 705 | 0 | 91 | 1 | 1.28 (0.62-2.67) |  |
| 19.6-20.7 (7,284) | 201 (3.3) | 6,120 | 18 (1.8) | 1,009 | 4 (2.6) | 155 | 1 | 0.50 (0.30-0.82) | 0.74 (0.27-2.03) |
| 20.7-21.6 (7,194) | 373 (6.4) | 5,854 | 65 (5.7) | 1,143 | 7 (3.6) | 197 | 1 | 0.87 (0.66-1.16) | 0.49 (0.23-1.05) |
| 21.6-22.4 (7,291) | 711 (12.1) | 5,894 | 92 (7.8) | 1,176 | 24 (10.9) | 221 | 1 | 0.61 (0.49-0.78) | 0.79 (0.51-1.24) |
| 22.4-23.2 (7,170) | 1,182 (20.5) | 5,770 | 141 (12.6) | 1,117 | 31 (11.0) | 283 | 1 | 0.57 (0.47-0.69) | 0.42 (0.29-0.62) |
| 23.2-24.0 (7,247) | 1,578 (27.0) | 5,841 | 233 (21.0) | 1,111 | 51 (17.3) | 295 | 1 | 0.72 (0.61-0.84) | 0.51 (0.37-0.69) |
| 24.0-24.8 (7,241) | 2,111 (36.4) | 5,807 | 320 (28.8) | 1,113 | 76 (23.7) | 321 | 1 | 0.71 (0.61-0.81) | 0.48 (0.37-0.63) |
| 24.8-25.8 (7,267) | 2,745 (47.2) | 5,814 | 419 (38.0) | 1,104 | 105 (30.1) | 349 | 1 | 0.71 (0.62-0.81) | 0.43 (0.34-0.54) |
| 25.8-27.8 (7,204) | 3,418 (58.4) | 5,851 | 491 (48.6) | 1,010 | 146 (42.6) | 344 | 1 | 0.69 (0.60-0.79) | 0.47 (0.37-0.58) |
| ≥27.8 (7,218) | 4,501 (75.1) | 5,995 | 614 (67.0) | 916 | 202 (65.8) | 307 | 1 | 0.77 (0.66-0.89) | 0.60 (0.47-0.77) |
|  | 16,873 (28.4) | 59,392 | 2,402 (23.1) | 10,404 | 646 (25.2) | 2,563 | 1 |  |  |

NAFLD, non-alcoholic fatty liver disease; BMI, body mass index

Data are numbers (%)

＊defined as doing physical exercise of at least moderate intensity more than 3 times per week, for at least 30 minutes each time, for an uninterrupted duration of at least 3 month at the time of the questionnaire

†Adjusted for age and sex. Estimated by binary logistic regression analysis
